# Supplementary material for: Amyloid-Beta Peptides and Activated Astroglia Impairs Proliferation of Nerve Growth Factor Releasing Cells In Vitro: Implication for Encapsulated Cell Biodelivery-Mediated AD Therapy
Source: Cells. 2021 Oct 21;10(11):2834. doi: 10.3390/cells10112834 (PMC8616486; doi:10.3390/cells10112834)
Supplement: Supplementary file 1 [file cells-10-02834-s001.zip › cells-1365113-supplementary.pdf]

Amyloid-beta peptides and activated astroglia impairs proliferation of Nerve Growth Factor releasing cells *in-vitro* - Implication for encapsulated cell biodelivery mediated AD therapy

Sumonto Mitra[1, *], Silvia Turchetto[2,3], Winant Van Os[2,4], Lars Wahlberg[5], Bengt Linderoth[6], Homira Behbahani[2,7, *], Maria Eriksdotter[1,8]

Karolinska Institutet, Department of Neurobiology, Care Sciences and Society (NVS), Center for Alzheimer Research - [1]Division of Clinical Geriatrics, [2]Division of Neurogeriatrics; [3]GIGA-Stem Cells and GIGA-Neurosciences, Interdisciplinary Cluster for Applied Geno-proteomics (GIGA-R), University of Liège, Belgium; [4]Wiskunde en Natuurwetenschappen, LIC/Chemical Biology, Leiden Institute of Chemistry, The Netherlands; [5]Gloriana Therapeutics, Inc., Providence, Rhode Island, USA; [6]Department of Clinical Neuroscience, Karolinska Institutet; [7]Karolinska University laboratories, Karolinska University Hospital, Stockholm, Sweden; [8]Theme Aging, Karolinska University Hospital, Huddinge, Sweden.

Materials and Methods

1. Cell death assay – As described in manuscript section 2.8, NGC0211 cells are exposed appropriately to Aβ$_{40/42}$ peptides or ACM's and stained with FITC-Annexin-V or propidium iodide (PI) and analysed in a flow-cytometer (BD Accuri C6). The presented data is showing cells stained both for Annexin and PI (Annexin V$^+$, PI$^+$), which are designated as late apoptotic cells.

2. Stress markers – To assess the extent of stress induced by Aβ$_{40/42}$ peptides or ACM's (astrocyte conditioned medium), redox imbalance (ROS, GSH) and mitochondrial stress (metabolic activity and mitochondrial membrane potential, ΔΨm) was measured as mentioned in manuscript section 2.6. Briefly, ROS (20 μM DCFH-DA), GSH (50 μM mBCL), metabolic activity (1 × alamarBlue) and ΔΨm (0.2 μM TMRM) was measured in NGC0211 cells after 24 h exposure, respectively.

3. Mitochondria staining – To evaluate mitochondrial network, 5×10^4 cells/well (for 24/48/72 h exposure) or 3×10^4 cells/well (7 days exposure) were seeded in Nunc Lab-Tek Chamber Slides (4 Well) and exposed to Aβ$_{40/42}$ (1, 0.1 μM) or equivalently treated ACM's, or left unstimulated. After 24/48/72 h, media was replaced with complete DMEM/F12 medium containing 500 nM MitoTraker Orange (Invitrogen) for 10 min, followed by 15 min incubation with dye-free complete medium. Cells were then washed 3× with PBS, before and after fixation in phosphate buffered formaldehyde 4% (v/v) (Sigma-Aldrich) for 5min at RT. Air dried slides were finally overlaid with mounting medium containing DAPI (VectaShield) and examined in an inverted laser scanning microscope (LSM 510 META; Zeiss).

4. Immunostaining – As described in main manuscript section 2.7. Briefly, post-exposure with respective treatments, cells were fixed (4% phosphate buffered formaldehyde, 5 min), permeabilized (0.2% Triton X-100, 15 min), blocked (1% BSA, 30 min) and probed with required primary antibodies as follows: mouse anti-human Ki67 monoclonal antibody; (1 : 700 dilution), mouse anti-GFAP monoclonal antibody (Biolegend, Cat no. SMI-26R); or 1 : 100 dilution of rabbit anti-S100 polyclonal antibody (Dako, # Z0311) overnight at 4°C. After 3× wash, cells were further incubated respective secondary antibodies. Cells were then washed, mounted using DAPI containing mounting medium and imaged.

5. Complement-C3 ELISA - C3 protein levels in the astrocyte-derived conditioned media were measured by using a human C3 Elisa Kit (Abcam, ab108823), as per manufacturer's specifications. Data were analysed by using the software SoftMaxPro.

6. ELISA – As described in manuscript section 2.9 using ELISA kit (R&D Systems; Cat No. DY256). Briefly, cell culture samples from NGC0211 cells grown in 2D or 3D conditions were treated with Aβ40/42 peptides or ACM's. Plates were coated with capture antibody, and after blocking with 5% BSA, samples were added to respective wells and incubated overnight. Plates were then washed and incubated with detection antibody. Following wash, substrate solution was added to each well and plate was read kinetically. Obtained data was normalized to a standard curve.

Results

1. Regulation of stress pathways in NGC0211 by Aβ40/42 peptides – Exposure of NGC0211 cells to respective stressors indicated inefficient alteration in cell death parameters (Figure S1A & 1B), along with marginal modulation of redox and mitochondrial stress markers (Figure S1C – 1F). Simultaneously, the mitochondrial network was initially hampered at 24 h but was efficiently restored by 72 h indicating the ability of NGC0211 cells to mitigate potential impact of stress factors (Figure S1G). These data show the resilience of NGC0211 cells to effectively counter Aβ40/42 peptide exposure for the time duration analysed in these experiments. There was no overall increment in cell death when exposed groups were compared to their respectively time-matched controls. The overall time-dependent increase in late-apoptotic cells could be an overall impact of cell density within the culture plate, which may induce physical stress.

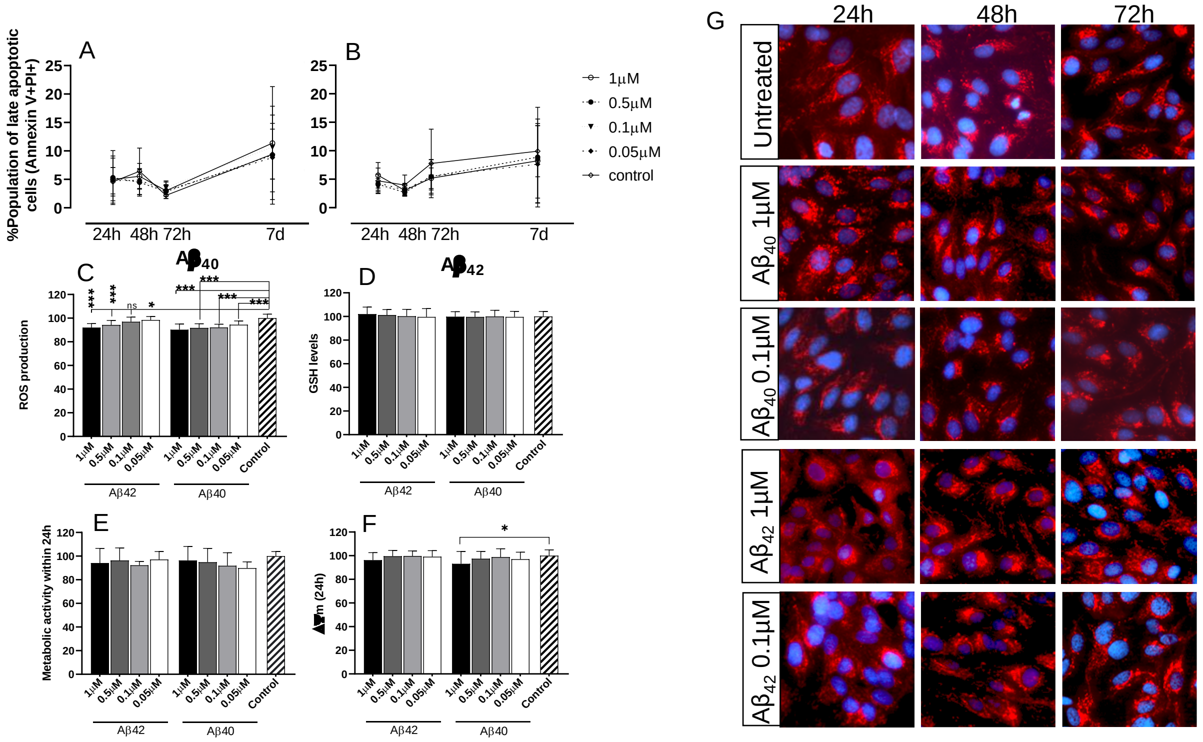

Supplementary Figure S1. NGC0211 cells were exposed to Aβ40/42 peptides for different time points and various assays were performed. (A-B) Post-exposure for 24 h, NGC0211 cells were harvested, stained with FITC-Annexin-V/PI dyes, and analysed in a flow-cytometer. Cells stained with both Annexin-V and PI were denominated as the cells undergoing late stages of apoptosis and are represented as percent of whole population gated. (C-F) Various stress

markers were evaluated after exposing NGC0211 cells for 24 h. ROS was significantly reduced with minor alteration in mitochondrial membrane potential (ΔΨm). (G) NGC0211 cells were exposed for 24/48/72 h and stained with Mitotracker Orange to evaluate the mitochondrial network within the cell. Data shows representative pictures of every time point. Data are represented as mean ± S.E. (N = 3). Statistical analysis using One-way ANOVA analyses with a Tukey's multiple comparison test was performed and compared to their respective controls. * = $p < 0.05$, ** = $p < 0.01$, and *** = $p < 0.001$.

2. Purity check of human cortical astrocytes along with their activation post-exposure with various stimulators (Aβ peptides, TNFα and IL-1β) – Purity of the astroglial cells were checked by their expression pattern of two welk known protein markers which are S100 and GFAP. This was followed by their ability to get activated after exposure with AD associated molecules utilized in this study (Aβ peptides, TNFα and IL-1β), and we found significant activation as assessed by GFAP staining and the morphological changes. Although heterogeneous in morphology, most of the control astrocytes displayed a polygonal shape, with few and short branches. Differently from the controls, treated astrocytes underwent process hypertrophy, as indicated by the higher number of branches. Interestingly, these morphological changes were more pronounced following Aβ treatment, compared to TNFα and IL-1β. Overall, we report that GFAP-positive astrocytes undergo treatment-specific morphological changes indicative of their reactive state, which were only partially detected in the control group.

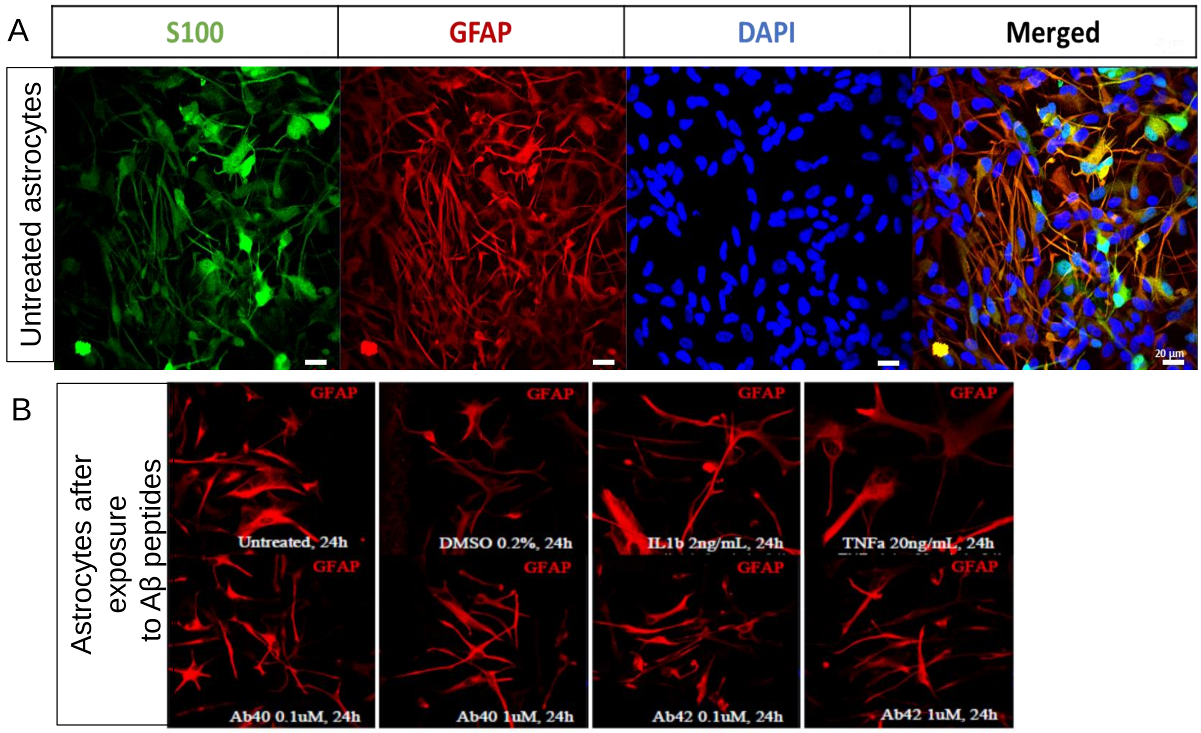

Supplementary Figure S2. (A) Purity checks of the astrocyte culture. To ascertain that the primary human astrocyte cells obtained from ScienCell Research Laboratories (Cat no.1800) were indeed pure astrocytes, we cultured the cells for 24 h and then immunostained with two

major markers of astrocytes – S100 (Green) (Dako, # Z0311) and GFAP (Red) (Biolegend, #SMI-26R). Dual staining shows that the astrocyte cells are indeed a pure culture. (B) Post-exposing astrocytes to A$\beta_{40/42}$ peptides (1, 0.1 μM), TNFα (20 ng / mL) and IL-1β (2 ng / mL) for 24 h in astrocyte culture medium, cells were stained with anti-GFAP primary antibody and visualized using an appropriate secondary antibody. The morphological features of the cells demonstrate their activated features post-treatment.

3. Impact of A$\beta_{40/42}$ peptides on release of complement-C3 and NGF release from astrocytes and hmNGF release from NGC0211 cells – It has been reported that Aβ and cytokine-induced astrocytes promote the complement activation, and that human RPEs constitutively express the C3-specific receptors. Since we observed astroglial activation following challenging them with various treatments of Aβ peptides and inflammatory molecules (TNFα and IL-1β), we wanted to check whether these astrocytes also release the complement protein (C3) and NGF (total levels). Using ELISA, we could ascertain that although TNFα (a well-known activator of astrocytes) could elicit C3 release from astrocytes, other treatment with Aβ peptides failed to show similar potency (Figure S3A). Similarly, previous reports indicates that astrocytes could be a source of NGF, and to make sure that these astrocytes originated NGF does not affect our estimation of NGC0211 released hmNGF levels, we estimated NGF release by astrocytes in ACM's after exposing them with appropriate treatments (Figure S3B). Surprisingly, Aβ peptides was found to upregulate NGF release from astrocytes, but the quantitative levels of NGF release by astrocytes were negligible in contrast to the amount released by NGC0211 cells (compared to Figure S3C). Likewise, to understand whether ACM exposure alters hmNGF releasing ability of NGC0211 cells, cells were exposed to ACM's for 24 h and hmNGF was measured from the supernatant (Figure 4C, main manuscript). As a follow-up, to check if there is a withdrawal long-term effect of ACM treatment, media was replaced from the treated wells and fresh media was added for another 24 h. Culture supernatant was again collected and analysed for hmNGF release (Figure S3C). We observed a slight increase in hmNGF released post-ACM exposure indicating positive modulation of NGC0211 cells by ACM's.

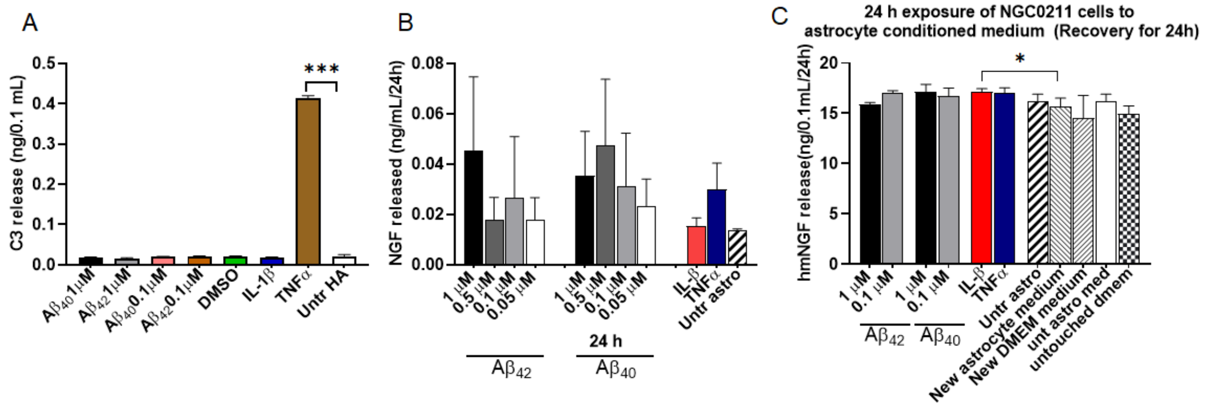

Supplementary Figure S3. (A) Astrocytes (7 × 10^4 cells/well) were cultured in 24-wellplate and incubated with Aβ peptides (1, 0.1 μM) and inflammatory molecules (TNFα and IL-1β) for 24 h. Culture supernatant was then collected and analysed using human complement C3 ELISA Kit (Abcam, ab108823), as per manufacturer's specifications. (B) From similar experiments, after exposing astrocytes to various treatments, cell culture supernatant was collected and analysed for released NGF levels using ELISA kit (R&D Systems; Cat No. DY256). (C) Post Aβ_{40ACM}, Aβ_{42ACM}, A$\beta_{IL\text{-}1\beta/TNF\alpha ACM}$ exposure to NGC0211 cells, the treated

ACM's were replaced with fresh DMEM/F12 medium and incubated for another 24 h. To set up the assay in a controlled manner, several control were utilized as follows – Untr astrocyte med– actual experimental control equivalent to untreated astrocyte media control in previous ACM experiment; New astrocyte medium and New DMEM/F12 medium – this group was utilized to evaluate the effect of culture medium on NGF release in previous ACM experiment; Untr astro med – the actual untreated ACM was not replaced with fresh medium; Untouched DMEM – the actual untreated NGC0211 cells plated in DMEM/F12 media was not replaced with fresh medium and allowed to grow for 48 h straight. These different control set-ups demonstrate the under various combinations of NGC0211 cell culture, the hmNGF release remains unaffected. Data are represented as mean ± S.E. (N = 3). Statistical analysis using One-way ANOVA analyses with a Tukey's multiple comparison test was performed and compared to their respective controls. * = $p < 0.05$, ** = $p < 0.01$, and *** = $p < 0.001$. Untr: Untreated.

4. **Regulation of stress pathways in NGC0211 by ACM's (astrocyte conditioned medium)** – To evaluate whether molecules release by activated astrocytes can modulate stress response from NGC0211 cells, NGC0211 cells were incubated with different ACM's for 3 h or 24 h and various parameters were evaluated. To control for the change in media composition, a group of NGC0211 cells growing in DMEM/F12 was kept as reference. Significant difference was observed when NGC0211 cells were incubated with various ACM's when compared to cells grown in DMEM/F12, but insignificant alterations were observed when treatment groups were compared to untreated astrocyte group, indicating the inability of Aβ peptides and inflammatory molecules to affect ROS, GSH, metabolic activity, mitochondrial membrane potential (@24 h) and mitochondrial connectivity (@3 h) in NGC0211 cells.

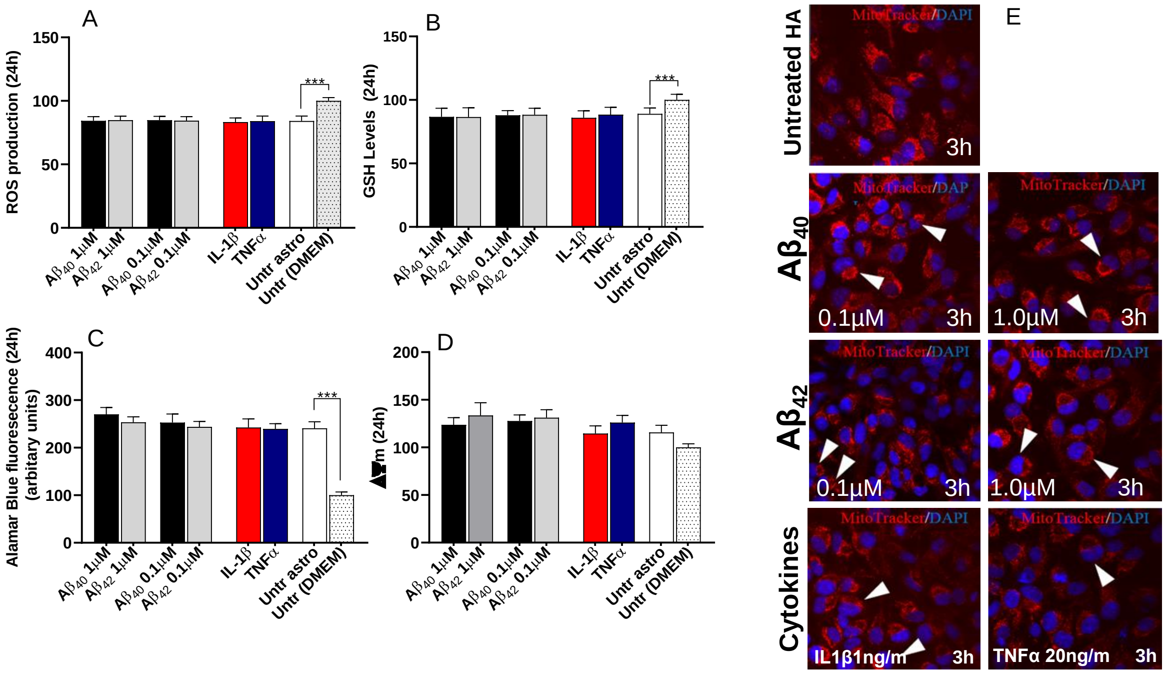

Supplementary Figure S4. – NGC0211 cells were incubated with ACM's and various stress parameters were evaluated. (A-D) Following 24 h incubation, ROS, GSH, metabolic activity and mitochondrial membrane potential was evaluated, respectively. Data are represented as mean ± S.E (N = 3). Statistical analysis using One-way ANOVA analyses with a Tukey's multiple comparison test was performed and compared to their respective controls. * = $p < 0.05$, ** = $p < 0.01$, and *** = $p < 0.001$. (E) Following 3 h exposure, cells were stained with Mitotracker Orange, and the mitochondrial network was visualized under a fluorescent

microscope. Data shows representative pictures of each group. HA: human astrocytes; Untr: Untreated.

5. Impact of Aβ40/42 and ACM's on NGC0211 cell proliferation - Both the inflammatory molecules (IL-1β and TNFα) showed anti-proliferative potential whether exposed to NGC0211 cells directly (Figure S5A) or via ACM's (Figure S5B) for different time points. IL-1β and TNFα reduced Ki67 expression from the early time point of 24 h and maintained this effect until experiment completion (72 h). Direct comparison with Aβ peptides (Figure 3, main manuscript) showed that the inflammatory molecules showed early and higher anti-proliferative effects, but the Aβ peptides showed severe time-dependent anti-proliferative effects which were higher at 72 h (than previous time points). When exposed directly, Ki67 expression was affected equally throughout all the points, but when ACM's were used, the anti-proliferative impact increased time dependently (especially for TNFα). The decreasing Ki67 expression observed in the control group can be explained by the fact that the proliferative capacity of the cells (growing in normal culture media) will be slowed with time due to the limited space availability and the cells reaching a confluent state.

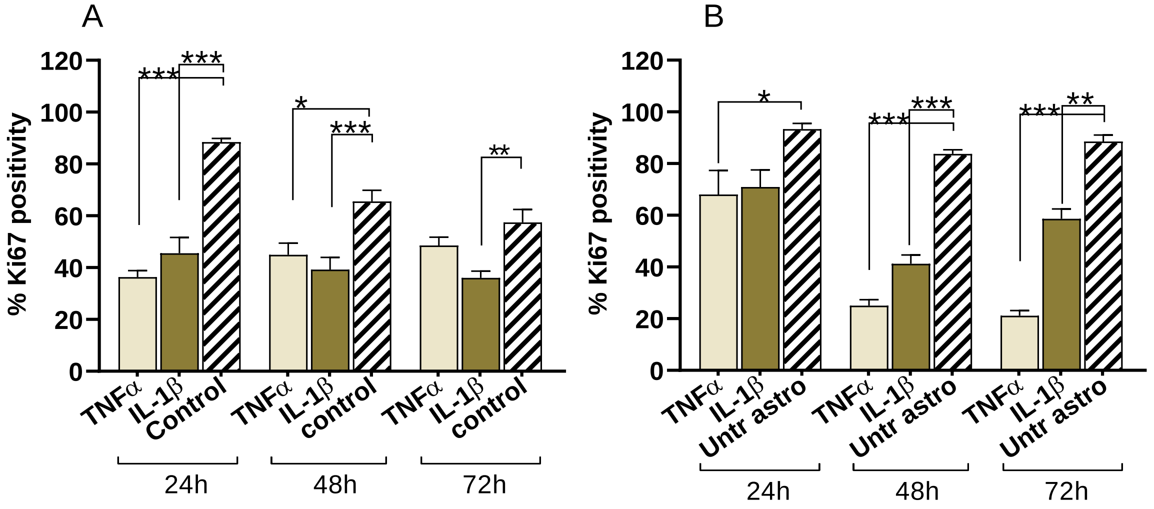

Supplementary Figure S5. Ki67 immunostaining shows anti-proliferative action of TNFα and IL-1β to NGC0211 cells. Cells were incubated (A) with interleukin-1beta (IL-1β, 2 ng / mL) or tumour necrosis factor alpha (TNFα, 20 ng / mL) directly, where control denotes unexposed NGC0211 cells; or (B) with $ACM_{IL\text{-}1\beta}$ and $ACM_{TNF\alpha}$ - for 24, 48 and 72 h where untreated astrocytes (Untr astro) describes the control samples which were exposed to ACM from untreated astrocytes (not activated). Ki67 immunostaining was performed, and data was acquired/analysed as mentioned in the manuscript. Briefly, Ki67 positive cells were counted, and percentage was calculated against DAPI stained cells (total cells in each analysed field). The percentage is represented here as % Ki67 positivity. Data are represented as mean ± S.E. (N = 3). Statistical analysis using One-way ANOVA analyses with a Tukey's multiple comparison test was performed and compared to their respective controls. * = $p < 0.05$, ** = $p < 0.01$, and *** = $p < 0.001$.

6. Impact of direct and indirect effect on metabolic activity of ECB devices – To evaluate whether the transfection with hmNGF releasing vectors altered the activity of ARPE-19 cells, differently transfected cells (untransfected ARPE-19, NGC0295 and NGC0211) cells were cultivated within ECB devices and their metabolic activity was measured after exposing them to Aβ peptides. The direct exposure groups demonstrate that the transfection process overall

reduced the metabolic activity in NGC0295, and further in NGC0211 cells, respectively. But when ECB-ARPE-19, ECB-NGC0295 or ECB-NGC0211 were exposed directly to Aβ peptides, there was no significant alterations when compared to control. Similarly, although no treatment specific changes were observed when ECB's were challenged with ACM's, but there was an overall increase in metabolic activity (when compared to direct exposure ECB's) which is similar for all types of ECB's (independent of the transfection process used to generate the cells – NGC0295 vs NGC0211), indicating it is a conserved response of the ARPE-19 cell type.

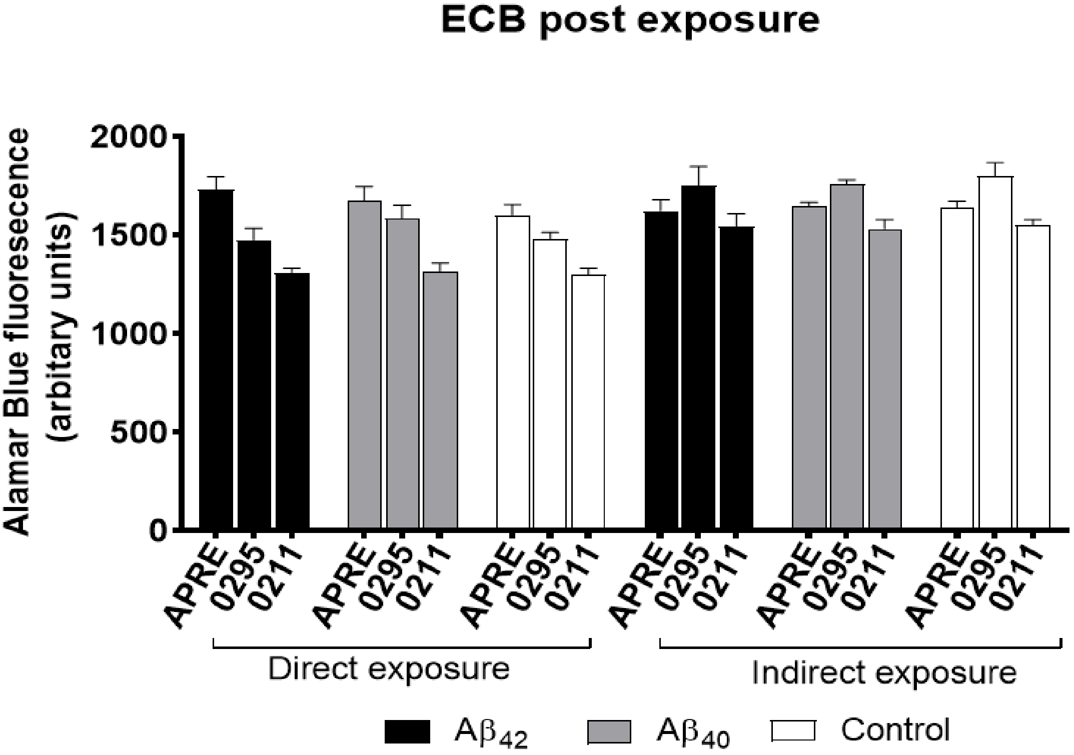

Supplementary Figure S6. – Comparison between different cell type carrying ECB devices - Following direct exposure ($A\beta_{40}$ and $A\beta_{42}$) or indirectly through treated astrocytes conditioned medium with $A\beta_{40}$ and $A\beta_{42}$, the metabolic activity of ECB devices was measured using alamarBlue for 1hr incubation. This data demonstrates the difference among various types of ECB devices carrying different type of cells – un-transfected ARPE-19 as control, transfected 1st generation NGC0295 cells used in our previous studies, and transfected 2nd generation NGC0211 cells used in our current study. We show that irrespective of the exposure routine, the metabolic activity of the cells inside ECB device is unaffected after 7 days of incubation. Moreover, the basal cell line ARPE-19 is also unaffected by the exposure and there is no additional effect due to the transfection procedure.
